# Golgi: Interactive Online Brain Mapping

**DOI:** 10.3389/fninf.2015.00026

**Published:** 2015-11-17

**Authors:** Ramsay A. Brown, Larry W. Swanson

**Affiliations:** Department of Biological Sciences, University of Southern CaliforniaLos Angeles, CA, USA

**Keywords:** connectome, brain mapping, interactive, neuroanatomy, API

## Abstract

*Golgi* (http://www.usegolgi.com) is a prototype interactive brain map of the rat brain that helps researchers intuitively interact with neuroanatomy, connectomics, and cellular and chemical architecture. The flood of “-omic” data urges new ways to help researchers connect discrete findings to the larger context of the nervous system. Here we explore *Golgi’s* underlying reasoning and techniques and how our design decisions balance the constraints of building both a scientifically useful and usable tool. We demonstrate how *Golgi* can enhance connectomic literature searches with a case study investigating a thalamocortical circuit involving the Nucleus Accumbens and we explore *Golgi’s* potential and future directions for growth in systems neuroscience and connectomics.

## Introduction

The nervous system is an evolved computing network that solves the challenge of organizing adaptive cybernetic behavior in animals (Swanson, 2000). The strategy for understanding this network as a biological system of the body is simple and dates to classical antiquity: accurately observe and describe the form of the system, and clarify its mechanism of action by functional experimentation (Swanson, 1995). Thus, a necessary but not sufficient prerequisite is to describe and display accurately the structural organization of the nervous system: what are the parts, what is the internal organization of each part, and how are the parts interconnected? This structural approach was put on modern footing at the macroscopic level in the 1660–1670’s by Thomas Willis, and at the microscopic level in the 1880–1890’s by Santiago Ramón y Cajal and others (Swanson, 2014). The contemporary revolution in structural neuroscience began around 1970 and was based on the development of experimental axonal circuit tracing methods combined with histochemical methods for the cellular localization of molecules with immunohistochemistry and hybridization histochemistry (Swanson, 1999).

Despite annual improvements in neuroanatomical atlases and connectomic techniques a complete architectural description of the macrolevel mammalian connectome remains elusive. This is to no fault of the connectomic community but instead reflects the sheer scale and complexity of assembling such a description. To aid in managing the complexity of the task, both in terms of human organization and data management, we turn to automation via neuroinformatics.

Neuroinformatics systems aid in collecting, storing, communicating, analyzing, and visualizing neuroscientific data (Arbib and Grethe, 2001). We identified four emerging trends from neuroinformatics and human-computer interaction that constrained *Golgi’s* design: (1) previous product validation from other neuroinformatics tools; (2) the experimental status of natural language processing and automated reasoning systems; (3) the growing corpus of biocurated reports from legacy literature; and (4) the adoption of high-throughput connectomic techniques.

Neuroscientists are accepting modern digital tools, such as the Brain Architecture Knowledge Management System (BAMS; Bota et al., 2005) and BrainMaps.org (Mikula et al., 2008) into their research workflows. Other tools similar to that we describe here, such as NeuroVIISAS (Schmitt and Eipert, 2012) and the Allen Institute Brain Atlas (Sunkin et al., 2013), seek to integrate neuroanatomical reference sets with connectomic data for consumption by researchers. But adoption of these types of tools has not stopped with expert workers in the field: software for crowdsourcing scientific exploration—even to layman—has demonstrated the value of building systems and User Experiences that encourage participation at different levels of expertise (Seung, 2013). These two trends signaled to us that researchers were becoming increasingly comfortable adopting new software techniques into their scientific workflows (and thus that a potential user base for *Golgi* existed) and that simple tools and intuitive interfaces for neuroscience can and should be designed.

Natural Language Processing (NLP) and Machine Learning promise to enhance the quality and throughput of semi-automated neuroscientific curation and exploration. The automated synthesis of systems-level insights about neural connectivity from primary reports is improving but still requires human oversight: the current state-of-the-art in NLP for automating curation remains in an experimental stage (Burns et al., 2008). Without doubt, neuroscience will be disrupted by tools that intelligently and automatically assemble disparate reports into cohesive lines of reasoning. For the immediate future, however, generating high-level insights about the brain from primary reports remains a distinctly human task. With this in mind, we designed *Golgi* as a human-friendly tool to help users connect primary reports into high-level understandings.

The corpus of legacy literature contains decades of connectomic reports, and informatics teams are curating (portions of) this corpus into neuroinformatics workbenches. The analysis of this curated data has allowed some groups to generate exciting novel connectomic insights from previously disparate reports (Bota et al., 2015). New data streams stem not only from curation, but also from new high-throughput techniques. Tools such as functional magnetic resonance imaging generate massive amounts of raw data even in a single set of experiments. Between the staggering data generated by these new techniques and the growing body of curated literature we identified the need for tools that simplify how researchers navigate a growing corpus of nuanced, sometimes conflicting assertions of connectivity.

In designing *Golgi* we sought to provide neuroscience with a tool that makes a task traditionally challenging for many users (synthesizing the interactions between multiple nuanced neuroscientific findings) easier by bootstrapping it to users’ innate spatial reasoning skills. This pressure led us to design *Golgi’s* User Experience around the framework of an interactive, two-dimensonal embryonic fate map of the nervous system. The utility of this map is immediately obvious: it is a flatmap of the central nervous system that displays all of its macrolevel parts in a topologically consistent way, so it can be displayed easily on paper or a computer monitor, much like the wall maps of the earth pioneered by Mercator.

## Methods

### Where did this Map Come From?

When the nervous system first becomes embryologically recognizable, it is a topologically flat sheet of ectodermal tissue called the neural plate (Swanson, 2012). As development progresses, the plate forms a tube and the walls of the tube progressively differentiate by forming a hierarchy of gray matter regions (analogous to countries on a map of the earth) interconnected by white matter tracts (like airline routes between national capitals).

**Figure 1:**
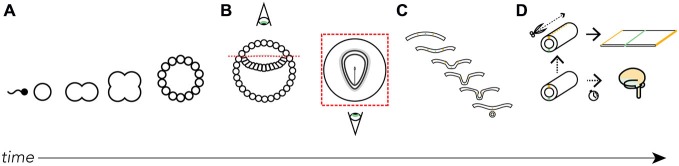
**Where did the map come from?** We derive the Swanson flatmap from embryological development. **(A)** After fertilization the newly formed zygote undergoes mitosis and early cellular differentiation. **(B)** After about 9 days (in humans) the ectodermal layer is distinguishable between a small (top) and large (bottom) cavity. With the amnion cut away and a top-down view of the ectodermal tissue we can differentiate the developing neural ectoderm from somatic ectoderm. This flat layer of neuroectoderm contains the neural plate, which generates all the progenitor cells that, with differentiation and division, will form the adult nervous system. **(C)** A cut view through the neuroectoderm over time reveals how the invagination of the once-flat neural plate forms the neural tube. **(D)** The neural tube undergoes cell division, tissue thickening, and corrugation at different rates and at different lengths along the tube’s long (rostrocaudal) axis throughout development. Should we imagine slicing along the long-axis of the dorsal surface of the tube we are able to unfurl it as we would a book. Here, we recreate the original conditions of the flattened neural plate. It is on this unfurled neural tube and its corresponding topological correspondence with the fates of each spatially-distinct cell line that we base the Swanson flatmap. For clarity certain features have been left out of the diagrams, including the inner cell mass **(A)** and endodermal layer **(B)**.

The adult “flatmap” that underlies *Golgi* can be conceptually formed by cutting the top (dorsal surface) of the neural tube lengthwise (rostrocaudally) and unfurling the walls of the tube like a book (Figure 1). The “fate map” part of the definition involves a series of assumptions based on a variety of evidence (both experimental and theoretical) about where adult nervous system parts are located in the neural plate—for example, the brain is located toward the head-end of the neural plate whereas the spinal cord is located toward the tail-end—and how they ought to be represented in two-dimensions given their volumes in the adult form (Swanson, 2004).

### Building the Map

The atlas displayed in *Golgi’s* UI (User Interface) is a subversion of the canonical rat embryonic flatmap featured in Swanson (2004) rat atlas.

**Figure 2:**
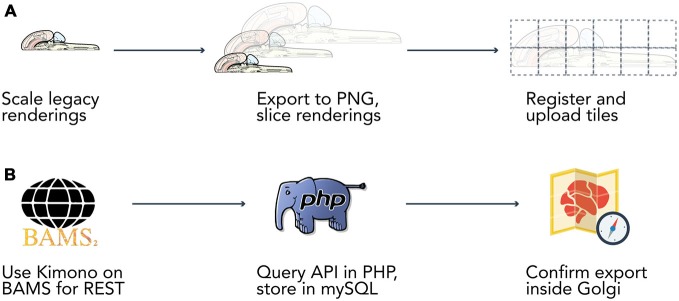
**Data extraction pipelines.** Two bespoke data extraction pipelines helped us process map data and information from the Brain Architecture Knowledge Management System (BAMS). **(A)** We built a semi-automated extraction pipeline for manipulating the Swanson (2004) flatmap into sizes and a format compatible with display on the web. We used Adobe Illustrator CS6 to modify the flatmap into three versions: a version only displaying the outline of the map, a version including the outline of each brain region, and a version that included abbreviation labels for each region. We then manually scaled each map version to four different resolutions (960 × 960, 1920 × 1920, 3840 × 3840, 7680 × 7680 pixels). Vector graphics were rasterized to 300 dpi Portable Network Graphics files and exported from Illustrator. We then used ImageMagick to automatically slice each map into 480 × 480 pixel tiles. Tiles were algorithmically named according to their scale and position for maintaining map co-registration. Brain regions and connections were isolated within Illustrator into their own files and saved as Scalable Vector Graphics files for display directly in browsers. **(B)** Data was extracted from BAMS with the use of the Kimono API (http://www.kimonolabs.com). BAMS follows a structured and predictable URI-based method for querying for data on brain regions, connections, molecules, and cell types but exposes no public REST API. This structure enabled us to use Kimono to expose a RESTful API representation of the data encoded on BAMS pages. Using PHP’s cURL library we then launched over 70k requests at our newly constructed REST endpoint, structured the response as to meet our data architecture, and stored the newly retrieved information in a series of MySQL tables.

Original vector graphics obtained from the author were modified using Adobe Illustrator CS6 on Mac OS X.10 to adapt the map for display in web browsers (Figure 2A) Once the map was transformed into a web-compatible Portable Network Graphic (PNG) format three versions were created: a version only displaying the outline of the map, a version including the outline of each brain region, and a version that included abbreviation labels for each region. These three versions were exported as 300-dpi PNG files at four levels of increasing resolution. Each of the four images was processed through a bespoke image processing pipeline powered by ImageMagick (ImageMagick Studio LLC, 2014) executed on Mac OS X.10 to slice the full-scale maps into constituent tiles for streamlined display on the net.

#### Frontend Design

*Golgi’s* frontend was designed to make it easy to query and visualize assertions of the mammalian nervous system’s anatomical, connective, chemical, and cellular architecture. It interprets as a series of hand-written HTML (Berjon et al., 2014) pages generated via PHP made interactive and styled by Javascript (ECMA Standard, 2011) and CSS (Etemad, 2011) resources, respectively. Publicly-available Javascript microlibraries such as jQuery (JQuery.com, 2012) were utilized to aid in (amongst other purposes) communication (via asynchronous HTTP calls), security (via the Secure Hash Algorithm), and display (via the jQuery UI library). To streamline design and ensure a consistent visual language throughout the product we used Twitter Bootstrap’s core CSS library for UI styling. All frontend code is available freely at https://github.com/ramsaybr/golgi. We encourage the community to fork, improve, and extend *Golgi* freely.

Here we explore key design decisions we made while developing *Golgi* with special attention paid to features that distinguish *Golgi’s* User Experience.

##### Unregistered Use

**Figure 3:**
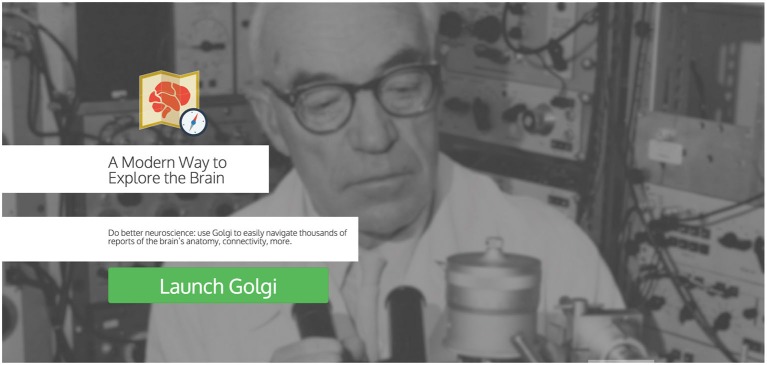
**Landing page.** *Golgi’s* landing page presents users with a clear Call to Action button for launching into *Golgi*. Scrolling down reveals an informative video and pages describing *Golgi’s* capabilities.

*Golgi* is free to use without first creating a user account, allowing users to immediately start using *Golgi* to explore the nervous system (Figure 3). This decision lowers barriers to adoption and help users investigate how *Golgi* could aid their research without requiring them to commit to a registration process.

##### Search

**Figure 4:**
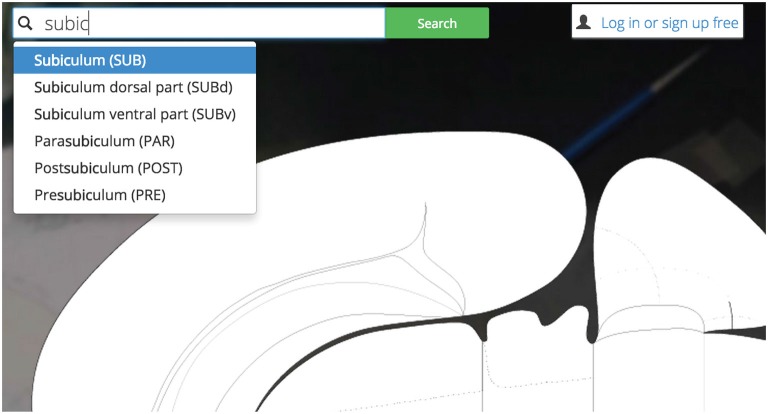
**Search bar.** *Golgi’s* auto-completing search box makes it easy for users to discover their brain region of interest and become familiar with the Swanson (2004) nomenclature.

Users may explore *Golgi* by first interacting with a search inputbox (Figure 4). The search inputbox lets a user look up a gray matter region (a node in network parlance) by either its anatomical name or its atlas abbreviation.

This feature both creates and solves a UI discoverability problem: new users or users familiar to alternative nomenclatures may not be familiar with the set of available Swanson (2004) brain regions they could search for (thereby limiting utility). To ameliorate this discoverability problem we implement a predictive text autocompletion algorithm. This will help experienced users access regions of interest more quickly and helps novice users learn the Swanson (2004) terminology more quickly.

Once a user begins typing, the autocompletion algorithm will suggest potential brain region matches. As a user continues typing, the potential match list will narrow until the user is presented with her correct region of interest. This is accomplished by pre-loading an array containing the names and abbreviations of all regions available and using the jQuery “TypeAhead” library for text autocomplete.

##### Inline Display of Visualizations Options

Once the user selects a region of interest she will be presented a summary of its available data within *Golgi* and will be prompted to “add” the region and highlight it on the map.

Highlighted brain regions are displayed and marked with a context-dependent “pin” icon on the map. The pin’s appearance depends on the set of region-associated data currently displayed on the map. This design makes it easier for users to understand what modalities of data are currently active on the map. For example, if a user has selected only connectional data about a region, the pin’s appearance will reflect this choice. A region displaying only molecular data will have a different pin, and a region with both active molecular and connectional data will show still a third type of pin.

**Figure 5:**
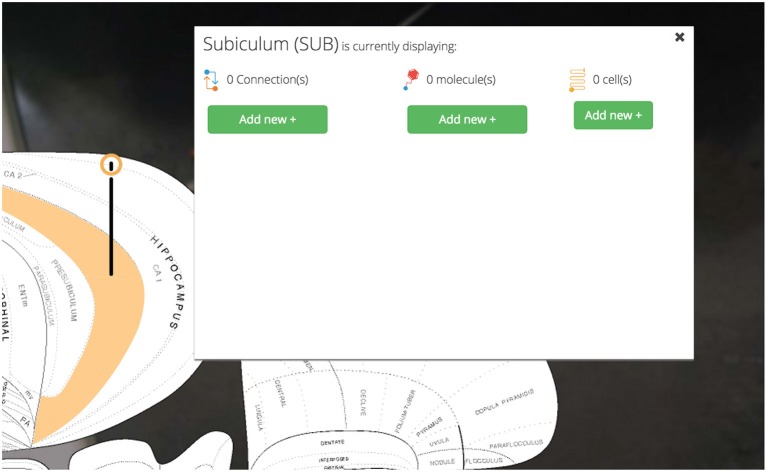
**Pins, visualization options.** User-selected brain regions are displayed and marked with a context-dependent “pin” on the map. The pin’s appearance depends on the set of region-associated data currently displayed on the map. Visualization options for displaying connectional, molecular, and cell-type assertions are displayed immediately adjacent to the selected region. This makes *Golgi* easier to use by cutting down on the amount of visual search a user has to perform.

Clicking a pin offers visualization options for assertions about connectivity, molecules, and cell types associated with the selected region (Figure 5). These options are displayed in a UI element dynamically placed adjacent to the region of interest on the map. Decreasing the amount of visual search a user will perform to navigate the interface will make it easier for users to select the information they want displayed.

##### Links to Primary Literature, BAMS

*Golgi* will help users more efficiently find primary sources that support assertions about neural architecture.

**Figure 6:**
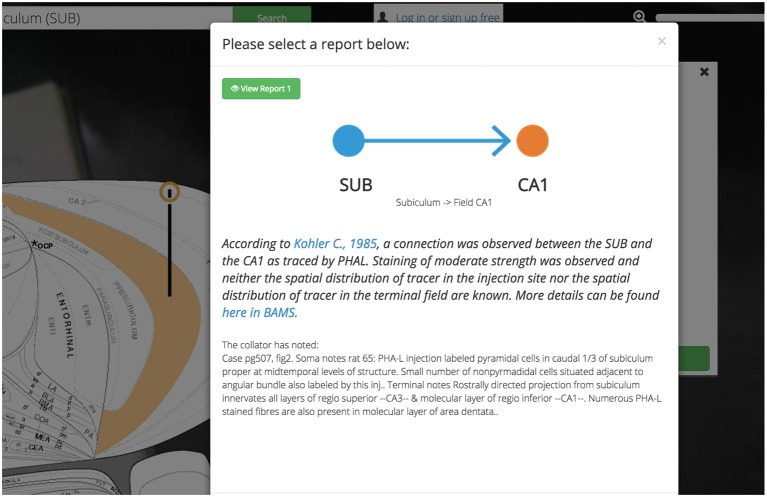
**Reports summary screen.** *Golgi* displays assertions about connections, molecules, and cell types in the brain. *Golgi* makes it easy to access the reports from the primary literature that underlie each assertion in *Golgi*. Each report contains a textual description of the report (including the experimental method used) a link to the original manuscript’s PubMed entry, and a link to the report in BAMS.

Expert-curated supporting evidence and links to primary sources are available for every assertion of connectivity and molecular and cellular architecture. Most assertions are backed by many reports sourced from the same or different primary sources. Many of these reports (to varying degrees) agree with one another, some do not. We present these reports, conflicting or otherwise, free from influence or normativity about technique or conclusion (Figure 6). This design decision was made to balance the efficiency of creating a User Experience based around assertions about the nervous system with the necessary respect for the oft-conflicting reports in the literature.

Each report associated with an assertion is available for inspection independently and is presented with a prosaic textual summary. These summaries read in plain-English and contain information such as the experimental technique used, the injection site in a pathway-tracing experiment, the relative strength and distribution of pathway tracer (if curated), and any notes left from the original curator. The decision to display this data as text (as opposed to in a table) was fueled by our desire to tutorialize new users on the nature of the assertions available in *Golgi*.

All assertions, and their associated connectivity reports, will present the user with not only a hyperlink to the PubMed report page for the cited primary source, but also with a link back to the BAMS connection details page corresponding to the data presented in *Golgi*. Both of these outbound-linking features will encourage curious users to not only engage with the larger system of BAMS but more deeply explore the data available in the primary source manuscript as well.

##### Data Layers

A selected region (and its associated assertions) is placed into a “Data Layer” when visualized to the map. Data Layers will let the user segregate assertions into distinct groups that can be selectively displayed or temporarily hidden on/from the map. Data can be moved between Data Layers and Data Layers can be created or destroyed.

Data Layers will help users compare data on the map. Keeping Layers isolated but visually co-registered allows researchers to build models while keeping some assertions distinct. For example, a user comparing the relationships between two connectional circuits that share a common node region will be able to keep each circuit isolated in its own Layer. She will be able to focus on one circuit by selectively displaying that Layer and hiding the other. Finally, she will be able to compare these two circuits with a third Data Layer that only contains molecular data for yet another region. Inspiration for this feature was drawn from analogous features found in graphic design programs like Adobe Illustrator and commercial mapping software like Google Maps.

**Figure 7:**
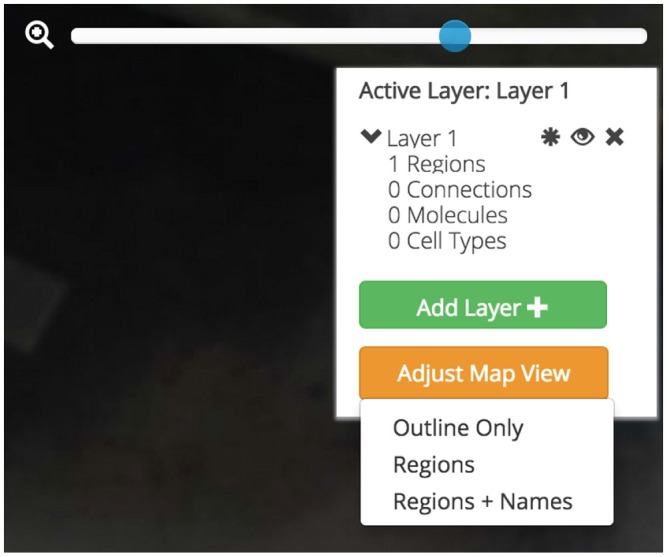
**Data layers, map visualization, and map control affordances.** Three different map visualizations can be displayed: a version only displaying the outline of the map, a version including the outline of each brain region, and a version that includes abbreviation labels for each region. Each map can be easily panned by clicking-and-dragging the mouse and zoomed using a zoom slider. Active brain regions and assertions can be selectively shown and hidden within Data Layers. Data Layers can be created, shown and hidden, and destroyed.

Users will be afforded control over the level of detail displayed on the flatmap (Figure 7). With the Map Layer affordance a user will be able to select from a visualization that only shows the gross outlines of the nervous system, another that includes the outline of individual regions, and a third that includes the Swanson (2004) nomenclature names of the regions superimposed. A click-and-drag interface affords an intuitive interaction point with the map and a familiar “zoom” scaling tool will allow users to enlarge the map to focus in on regions of interest without compromising map quality.

##### Account Personalization

**Figure 8:**
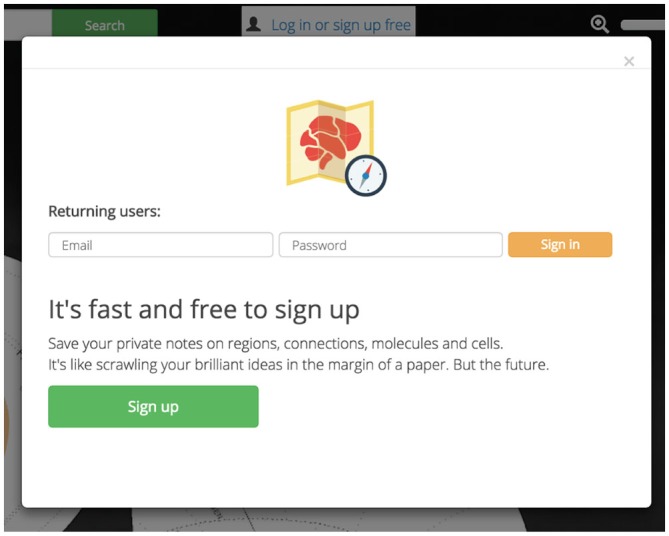
**Account personalization.** *Golgi* accounts are free and easy to sign up for. Accounts let users save personal notes on regions and assertions.

Private accounts personalize and enhance *Golgi*. *Golgi* accounts will allow users to save notes on any assertion for later use. This will let users easily record their thoughts, processes, and observations for later analysis or retrieval from a different computer (Figure 8). User data and notes are kept private between *Golgi* and each user.

##### Tutorialization

Design Thinking and user-testing in the development process help product designers and developers build tools that are intuitive to use and minimize barriers to consumer adoption. Yet despite best efforts, most designed systems (*Golgi* included) still benefit from explicit tutorialization. As such, we are developing a series of short, video-based “real life” examples of how *Golgi’s* features can be used. These videos will demonstrate the way users can interact with different parts of *Golgi* in the context of a typical use case. This will not only help instruct users in how to use Golgi, but also in why to use *Golgi* to solve real neuroscientific problems.

These videos will overlay directly onto *Golgi* (to place the explained behavior in context) and will be available via an outbound link to YouTube as well. They will provide examples of how to search for a brain part, activate a region on the flatmap, use the pins to explore afferent and efferent connectivity, explore evidence underlying an assertion of connectivity, and work with layers.

#### Backend Design: REST-First Neuroscience

*Golgi’s* backend is a RESTful (Fielding and Taylor, 2002) API custom-built from Apache2, PHP and MySQL. MySQL was chosen as our relational database management system because of its compatibility with our imported legacy data sources, and the authors had existing experience building and maintaining MySQL services. As an interpreted language PHP let us quickly test and evaluate our design decisions and rapidly deploy changes to the system, minimizing delays between developing and testing phases. Apache2 was selected as our HTTP server for its existing support within our Ubuntu staging and production servers and because it will adequately meet the usage demands of the product outlined here.

Adhering to a REST-first architecture promotes modular and reusable code, makes *Golgi* easier to maintain and grow, and will allow other members of the neuroinformatics community to use *Golgi* as a data source for their own projects.

For example, information about the Nucleus Accumbens is available over HTTP via a REST endpoint.

**Figure 9:**
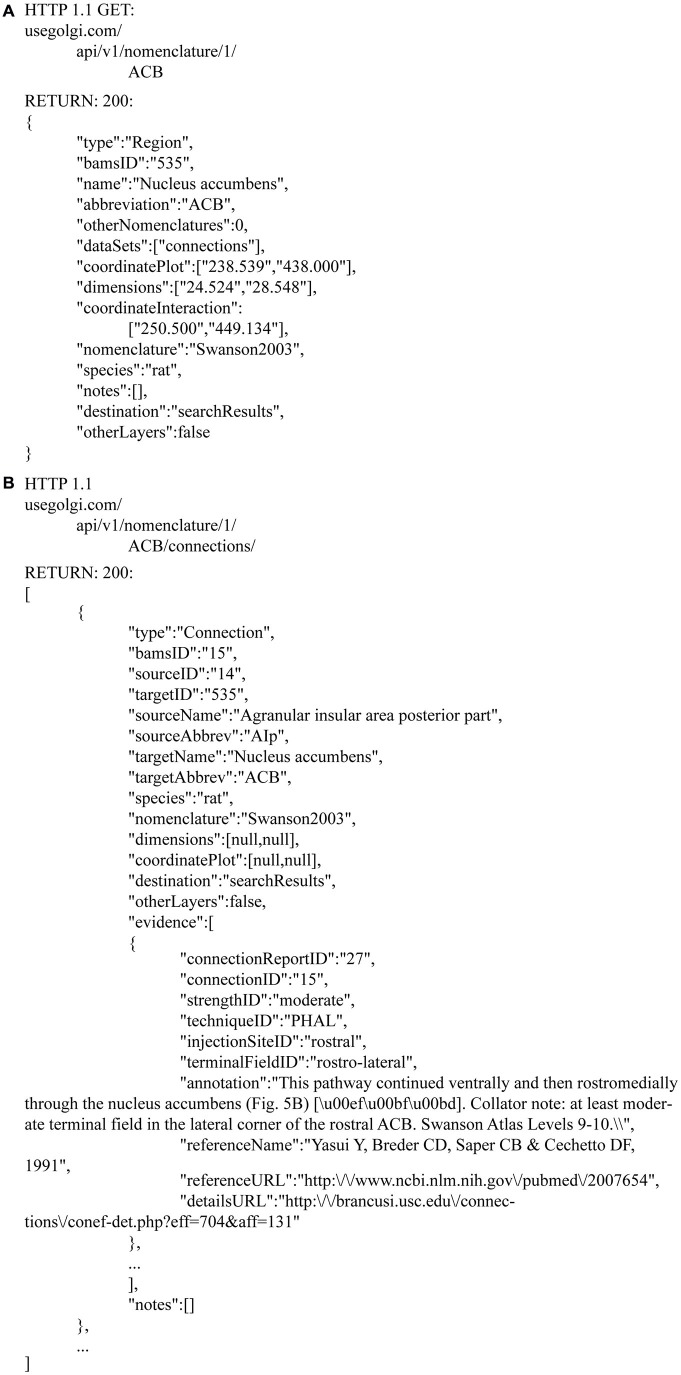
**JSON responses from the Golgi API.** The *Golgi* API returns JSON formatted responses to queries about brain regions, connections, molecules, and cell types. As an example we include here the JSON output describing **(A)** the Nucleus Accumbens and its **(B)** output connectivity. This response is generated from a request to http://www.usegolgi.com/api/v1/nomenclature/1/region/ACB. Responses are stateless and consistent in structure. Statelessness and structural consistency allows *Golgi* (and developers building on top of *Golgi* as a backend data service) to automate follow-on queries. For example, a request to the /region/ACB endpoint can be followed-on by requests to the /region/ACB/connection, /region/ACB/molecule, and /region/ACB/cell endpoints.

This endpoint returns JSON-formatted (Bray, 2014) information about the ACB (Figure 9). The response contains information both specific to *Golgi* (for example, coordinates for display on the Swanson, 2004 flatmap) and of general use to developers wishing to build their own neuroinformatics products powered by *Golgi*.

For example, the response.dataSets[ ] array contains information about what other data one can find associated with this region. In the Nucleus Accumbens example this includes macroconnections involving the nucleus (gray matter region). Because this data is returned in structured, predictable ways, it is amenable to automated incorporation in follow-up requests to *Golgi’s* API.

A returned Connection object contains both source and target region data identifiers unique to *Golgi* as well as textual region names and abbreviations and information about the species and nomenclature for which these assertions are valid.

RESTful architecture lets developers consume *Golgi’s* as an API service layer. For example, future neuroinformatics tools will be able to take advantage of *Golgi*’s data about connectivity, chemical, molecular, and anatomical architecture via standard HTTP calls. This makes *Golgi* useful as a platform layer for the general neuroscientific community.

### Data Extraction for Map Content

*Golgi’s* data is sourced from the Brain Architecture Knowledge Management System (BAMS). BAMS’ databases of neuroanatomical parts, connections, cell types, and molecules provide *Golgi* with a large, expertly curated source of neuroscientific data. The parts, connections, cell types, and molecules encoded in *Golgi* and extracted from BAMS are species-exclusive to the rat. The decision to constrain *Golgi* to rat-based data was multifaceted. First and foremost BAMS contains more data about the rat than it does any other species. Second, Swanson’s hierarchical, internally-consistent nomenclature and flatmap either only exist for the rat (in the case of the nomenclature) or are the most detailed in the rat (in the case of the flatmap, for which he has also developed simpler, experimental flatmaps for other non-rat species). Finally, while inter-species neurohomology and comparisons of connectivity are a compelling line of inquiry in their own right, they were not our primary design goal for this product. As such, we have scoped *Golgi’s* current data offering to the rat, based on the Swanson (2004) nomenclature. Data extracted from BAMS was transformed from raw XML to records in MySQL inside *Golgi’s* API via a bespoke extraction pipeline written in PHP and powered by the Kimono API (Figure 2B). Data was then manually inspected for both completeness (all data that was desired was transferred) and transfer accuracy (what was represented in BAMS is faithfully represented in *Golgi*) once inside *Golgi’s* MySQL database. Development continued only once the extraction pipeline achieved both transfer accuracy and completeness.

The data visualized on *Golgi* are assertions of connectivity (or cytoarchitecture or chemoarchitecture), not individual reports of data as can be found in BAMS. This distinction is significant because it affects how users understand what *Golgi* is showing them (see “Links to Primary Literature, BAMS” Section). A connection that a user plots on *Golgi* is a summary of connectivity that has been supported by at least one report of connectivity curated from the literature.

### Development Technology and Production Infrastructure

**Figure 10:**
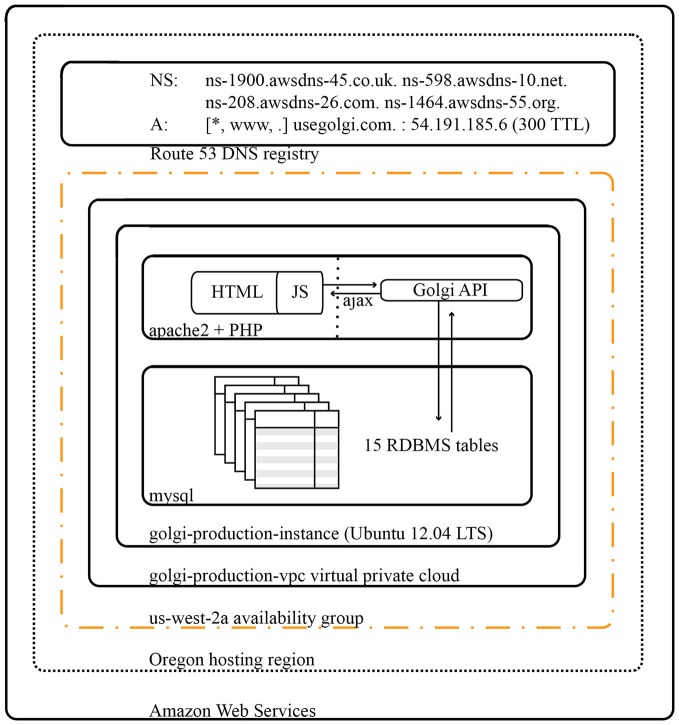
**Architectural diagram for *Golgi* system.** As a web app *Golgi* exists as a series of both frontend and backend PHP scripts with associated MySQL services for database storage. All scripts and services are executed on a 64-bit Ubuntu 14.04 LTS virtualized Elastic Cloud Compute instance on Amazon Web Services. The *Golgi* production instance is protected by its own virtual private cloud that is itself contained within the us-west-2a availability group out of Amazon’s Oregon computing facility. Amazon Elastic Cloud Computing’s demand-based elastic scaling minimizes the costs of operating *Golgi* while maximizing its performance.

*Golgi* is deployed on a virtualized 64-bit Ubuntu 14.04LTS Elastic Cloud Compute instance on Amazon Web Services (Figure 10). Amazon Elastic Cloud Computing’s elasticity minimizes the costs of operating *Golgi* while maximizing its performance. DNS Routing is managed via Amazon Web Services’ Route 53.

*Golgi* was developed on physical testing environments running Mac OS X.10 and Ubuntu 14.04LTS using Sublime Text 3, MAMP, and Ubuntu’s native LAMP stack for development and local testing. *Golgi’s* source code has been made available at http://www.github.com/ramsaybr/golgi. We encourage the community to fork, improve, and extend *Golgi* freely.

*Golgi* was developed and tested using Google Chrome’s Blink CSS layout engine and V8 Javascript engine. The extension of support for Firefox and Internet Explorer will require nontrivial code refactoring. First, client-side browser identification via Javascript will automate the detection of the user’s browser type and load the appropriate CSS and Javascript files optimized to that browser. *Golgi’s* current CSS, tested and optimized for the Blink layout engine, will be re-written and re-tested for both the Gecko (supporting Firefox) and Trident (supporting Internet Explorer) layout engines as to ensure a consistent User Experience across all browsers (particularly when Document Object Model [DOM] objects and their CSS properties are directly modified from Javascript). All of *Golgi’s* Javascript will also be re-tested and refactored as needed as to maintain consistency in user interactions, 3rd party library functionality (for example, with jQuery), and asynchronous communication with *Golgi’s* API between the V8 (Chrome), Chakra (Internet Explorer) and SpiderMonkey (Firefox) Javascript engines.

While future development priorities include extending support for Internet Explorer and Firefox browsers, we suggest that, for best performance, users access *Golgi* using Google Chrome.

## Results

### The Flipped Perspective of Model-First Inquiry

*Golgi* will let users explore connectivity assured that the primary literature supports them at each step of their model building. In designing *Golgi’s* User Experience around connectivity assertions (as opposed to reports from the primary literature) we promote a “model-first” paradigm for exploring connectomics. We bridge this assertion-level User Experience with the imperative for evidence-based reasoning by providing users an easy way to view details about the individual reports that underlie the connectivity assertions. Bolstered by links to the primary source from which the connection report was curated, this approach will enhance a user’s ability to quickly explore connectomic assertions without sacrificing scientific integrity. Once a user activates a connection she will be able to investigate the associated primary sources to better understand the data underlying their assertions.

This approach is a strength of *Golgi* as a research tool: a user will be quickly and easily build high-level connectomic models assured that the circuits she has outlined are supported by curated reports from the primary literature (as opposed to doctrine or authority). By reversing the literature search workflow (“model-first” as opposed to “source-first”), *Golgi* will provide her a way to quickly assemble high-level models of regions of interest and their systems-level relationships while respecting the imperative for source authenticity.

### Exploring the Nucleus Accumbens

*Golgi* combines the power of neuroinformatics databases with the usability and explanatory power of anatomical atlases. It will enhance traditional literature searches by letting a user start from a “model-first” perspective and focus-in on specific primary sources as she progresses. Here we demonstrate how this functionality will assist in model building by outlining a macrolevel circuit implicated in the hedonic response, addiction, and depression (Thompson and Swanson, 2010).

**Figure 11:**
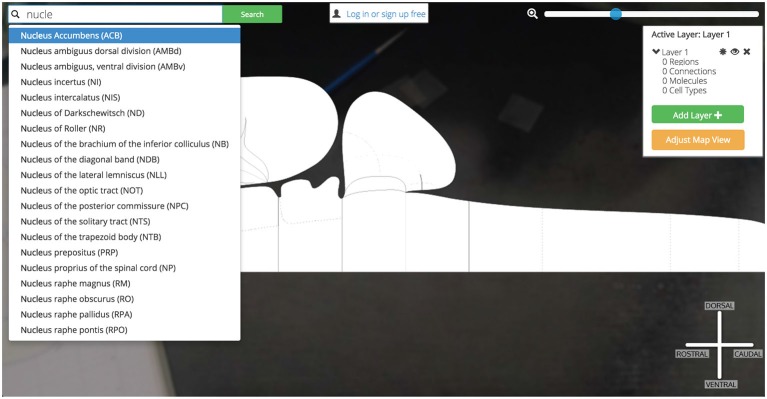
**Searching for Nucleus Accumbens.** Searching for a region of interest, like the Nucleus Accumbens, is made easier by *Golgi’s* autocompleting search algorithm. All possible matching options are displayed and subsequently removed as the user continues typing. This focuses the list down to the best possible matches to the region of interest.

We begin by searching *Golgi* for the Nucleus Accumbens: a small cerebral gray matter region located ventral to the dorsal Striatum (Caudoputamen) implicated in the hedonic response, addiction, and emotional valence. To find the Nucleus Accumbens we begin by using the search tool and starting to type in its name (Figure 11).

Before we have completed the term “Nucleus”, *Golgi’s* TypeAhead algorithm has constrained the available search results presented to us. The Nucleus Accumbens is displayed as a potential match. As we continue typing the search result is singled out as the only appropriate match to our input. We click the name and the green “Search” button.

Our search results (a summary of the data available about the Nucleus Accumbens) slides into view and we are presented with options for data visualization: we can display the Nucleus Accumbens on the map, we can display the Nucleus Accumbens on the map and immediately begin displaying associated assertions, or we can record notes about the Nucleus Accumbens that will save to our *Golgi* account.

As we click the green “Add ACB to map” button the search results slide out of view and the area on the map representing the Nucleus Accumbens highlights and changes color to amber. For clarity and to cue the user to their next behavior a pin is placed on the region. The pin’s crowning halo is a thin ring of amber to signify that the region is active but no assertions are currently displayed.

To clarify the anatomical context of the Nucleus Accumbens we adjust our map display options. We click the orange “Adjust Map View” button in the map control panel and select “Regions + Names”. This updates the map to include the anatomical boundaries between regions as defined in Swanson (2004) and labels each region with its neuroanatomical name (or abbreviation). We use the zoom slider to investigate more closely by zooming to an 800% enlargement. By clicking and dragging on the map we pan over to view the Nucleus Accumbens and the rest of the Striatum.

**Figure 12:**
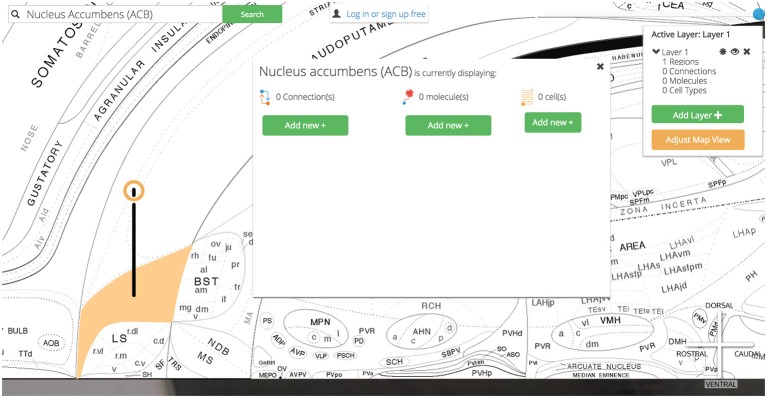
**Visualization options.** Once the region of interest has been found it is easily activated on the map. Activating a region colors it amber and drops a context-dependent pin onto the region. Clicking the pin displays visualization options (for the display of connection, molecular, and cell-type assertions) immediately adjacent to the pin itself. This helps streamline the user’s workflow by reducing the amount of visual search they have to complete.

Clicking on the Nucleus Accumbens’ pin reveals a UI element that summarizes the assertions currently displayed and our options for displaying more (Figure 12).

To begin our exploration of Nucleus Accumbens circuitry we click the green “Add new +” button underneath the “0 Connections” header. The current visualization summary is replaced with a panel for selecting input (afferent macrolevel connections terminating in the ACB) and/or output (efferent macrolevel connections emanating from the ACB) connections to visualize on the map. Here we select one input and two outputs: an afferent input from the Infralimbic Area (ILA) and two efferent connections to the Substantia Innominata (SI) and the Anterior Lateral Hypothalamic Area (LHAa). Once these regions have been selected we click the green “Add Selected to Map”button.

**Figure 13:**
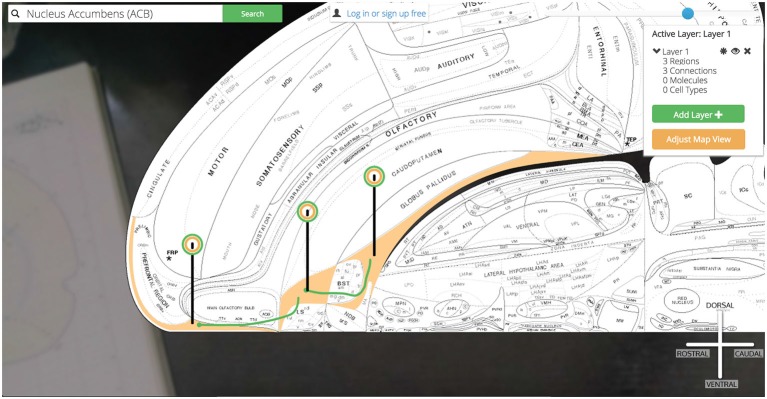
**Show three connections.** A simple multi-region circuit traced involving the Nucleus Accumbens and its connectional partners.

*Golgi*: (1) checks the browser’s memory for information stored on the ILA, SI, and LHAa and recalls it for displaying the regions properly; (2) downloads the image files that display those regions on the map and displays them according to data retrieved in step 1; (3) calls the *Golgi* API for connectional, molecular, and cellular assertions (and all supporting evidence) associated with these new regions [to speed-up future searches that the user is likely to make]; (4) downloads the image files encoding the connections that will be rendered on the map and displays them according to data recalled in step 1; (5) modifies the pin dropped on the ACB to include a green halo as well signifying that the ACB currently is displaying associated connectional assertions; and (6) drops similar pins on the newly displayed ILA, SI, and LHAa (Figure 13).

We have explored a simple macrolevel connection chain of (using the Neural Systems Language notation) @ILA >+ ACB @ACB >− SI, LHAa (Brown and Swanson, 2013). From here we continue our circuit building in the same manner. By clicking the pin for the SI we select an output connection to the LHAa and PT and add them to the map. Likewise we can select the LHAa pin and activate output connections to the PT as well.

**Figure 14:**
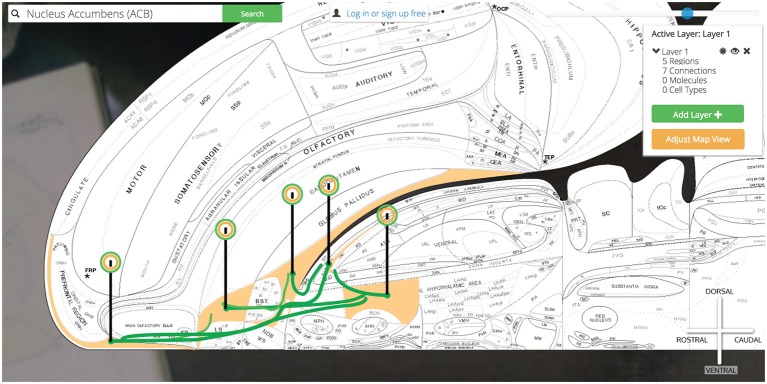
**Show all six connections.** A more complicated multi-region circuit is traced that explores some common connectomic motifs: connective convergence, divergence, and cycling.

Now our simple @ILA >+ ACB @ACB >− SI, LHAa has grown to @ILA >+ ACB @ACB >− SI, LHAa @SI >− PT @LHAa >+ PT as we have introduced a connectional divergence (@ACB >− SI, LHAa) and convergence (@SI >− PT @LHAa >+ PT). As we continue to plot output connections by selecting the PT we find the ILA as an available output target and select it for plotting (Figure 14).

We have traced a full thalamocortical loop between the ventromedial prefrontal cortex (ILA), a hedonic behavior modifier (the ACB), a behavior releaser and hypothalamic central pattern generator (the SI and LHAa, respectively) and a thalamic feedback relay (PT). *Golgi’s* interface for interacting with high-level assertions of connectivity made this task straightforward and helped us quickly model a nuanced macrolevel circuit.

**Figure 15:**
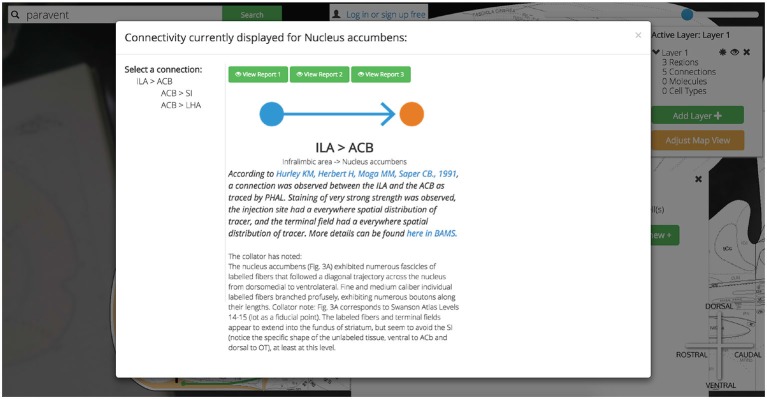
**Show ACB report from PNAS paper.** *Golgi* balances the constraints of ease-of-use with scientific rigor. Allowing users to explore the brain “model-first” and assemble connectomic models from assertions of connections helps them more quickly explore new possible combinations of circuits. Every assertion is supported by expert-curated reports from the primary literature. Here we show an example of a report from the Infralimbic Area to the Nucleus Accumbens. This report provides a high-level textual summary of the nature of the connection observed and the experimental parameters used to discover it. A link out to the PubMed reference and this report’s entry in BAMS is also provided.

To explore the reports supporting any of these assertions we need only click on any pin in the circuit. Where we previously used the data visualization interface to activate regions and connections to the map, here we use the “View Connection Details” button to learn more about the connections we visualized. All input and output connections involving the selected region of interest (in this case the ACB) are available for closer inspection. As we select the ACB >SI connection we are presented with a high-level summary of the connection, options for viewing individual reports supporting the connection collated from the literature, and a space to save notes on this connection to our *Golgi* account (Figure 15).

Clicking any of the green “View Report” buttons displays a summary of the primary document from which this connection report was sourced (including a hyperlink to the primary document’s PubMed entry) and where available, curated details describing the experimental technique.

## Discussion

Neuroinformatics offers tools that help researchers collect, analyze, store, and disseminate models and data. We note here two compelling patterns that emerged as the field grew.

First, frameworks emerged that promote structured exchange of information between neuroinformatics solutions (the federation of tools was not ensured as a natural consequence of the software ecosystem or development processes). Nevertheless the emergent pattern is often mutually beneficial to participating tools: federation and synthesis help each tool become more useful (and, by proxy, better adopted).

A second pattern emerged in which individual tools synthesize disparate modalities of neuroscientific data and assertions into a single User Experience. This trend—and tools leading it—are not new: systems such as the BAMS offer researchers these types of multifaceted solutions. Tools in this pattern (of which we have aspired to build *Golgi* as one) combine reports from multiple lines of neuroscientific inquiry in a common data framework (such as internally-consistent, hierarchical neuroanatomical nomenclatures and associated atlases) and provide users with multifaceted reports that help them connect disparate observations and data sources into systems-level models.

We see tools that follow this second pattern, that aggregate data from disparate lines of inquiry, holding the potential to enhance workflows for three user segments: (1) research investigators; (2) computational modelers; and (3) clinicians.

### *Golgi* can Facilitate Systems-Level Investigation

The state of the art in experimental neuroscience requires increasingly multifaceted experimental manipulations at multiple levels and scopes. For example, investigators designing optogenetic experiments often must incorporate data about neuroanatomy, cytoarchitecture, genetics, proteomics, and connectomics to properly execute their experiments. More challenging still: the multifaceted data they integrate should be internally-consistent in its method and anatomical framework. To this extent, there is a pressure for tools that help investigators seamlessly aggregate reports on disparate neurological phenomenon in an internally-consistent manner. *Golgi* will help investigators generate high-level insights within internally-consistent frameworks. This will facilitate new experiments better than will either isolated single-focus neuroinformatics tools or traditional literature searches.

### *Golgi* can help Facilitate Large-Scale Simulation Frameworks

Computational neuroscience has, on two ends of its spectrum, historically focused on emulating large biological-inspired neural networks with high levels of abstraction or simulating restricted ensembles of neurons with higher degrees of biological realism. Contemporary investigations like those outlined in the Human Brain Project aim to synthesize biological realism with large-scale networks of simulated neurons. This signals to us an increased pressure for tools that make biological neural network architecture readily accessible.

As semiconductor price-performance doubling increases, more research teams may find it feasible to incorporate large-scale biologically-realistic simulations into their experimental workflows. Tools like *Golgi* that provide streamlined access to aggregated connectomic findings may be positioned to provide the connectomic framework for neural simulation suites. The export of selected neural architectures or direct federation with computational simulation tools will offer the computational neuroscience community easy ways to incorporate structurally-observed network architectures into their simulations. To this end we predict that tools that streamline the exploration and interaction of connectomics data will experience closer relationships with computational neural simulation suites. In this scenario data exchange and system integration efforts can be expedited by bootstrapping APIs off existing interchange systems such NeuroML (Gleeson et al., 2010).

While artificial neural network simulation suites exist (Aisa et al., 2008) that simulate the macrolevel connectivity of layers constrained to behave as unique brain regions, some more sophisticated systems seeking greater biological plausibility require the specification of mesolevel connectivity (connectivity between distinct cell populations) (Arbib, 2003). To this end, *Golgi* would need to evolve to incorporate both macrolevel connections and mesolevel connections as well. In the near future connectomics may converge on both experimental techniques well-suited to mesolevel investigation as well as palatable ontologies for describing mesoconnectivity. So too will *Golgi* need to evolve to incorporate this new information and federate it with other neuroinformatics and simulation tools.

However, in its current form, *Golgi* contains information that relates brain regions to distinct cell types that have been reported within those brain regions as recorded in BAMS. Nevertheless, the data available in this iteration of *Golgi* is limited to reporting the presence of these cell types and does not encode any mesoconnectivity. This is due to two related reasons. Primarily, both the available techniques for exploring mesoconnectivity and consensus understanding of how to discuss and organize acquired connectional data at the mesolevel remain developmental (Bohland et al., 2009). Because of this, the connectomic data available online in internally-consistent, hierarchical mesolevel ontologies is sparse. As such, we restricted the scope of data included in *Golgi* to macroconnectivity.

### *Golgi* can Connect Benchside with Bedside

When tools are built with ease-of-use as a design constraint they maximize the scope of their potential user base. Balancing *Golgi’s* academic integrity with its ease-of-use was a primary design goal. The resulting User Experience minimizes barriers to adoption and encourages non-research experts to use it. Making *Golgi* easier to use for users like clinicians encourages its use as a dissemination and teaching platform.

In this scenario, neurologists and other clinicians will benefit from streamlined access to contemporary connectomics. Users in this segment often suffer from constraints of time, background understanding, and institutional access barriers for literature searches about a neural pathway of interest to be effective or efficient. We designed *Golgi* to minimize the amount of time or background knowledge a non-research expert will need to learn more about a particular pathway or circuit under investigation. By lowering these barriers to access *Golgi* can help clinicians maintain a contemporary understanding of neural architecture quickly and easily. Lowering these barriers to access for this user group may help improve outcomes as more clinicians are equipped with more current knowledge that helps them make better intervention decisions.

### *Golgi*’s Context Among Analogous Neuroinformatics Tools

*Golgi’s* development was encouraged by the existence and spread of other anatomical neuroinformatics frameworks. These other tools not only bolstered our belief that a tool like *Golgi* would be useful for the field, they provided both inspiration and a confirmation of how *Golgi* could distinguish itself from other tools and add value to researchers’ workflows. Here we briefly explore three analogous neuroinformatics solutions, their strengths, and how *Golgi* may distinguish itself from each.

NeuroVIISAS offers an open framework for storing, processing, visualizing, and simulating neural data. While the majority of data collected and available from the creating team at the University of Rostock focuses on the rat and mouse, neuroVIISAS is species-flexible. As an expert-oriented system neuroVIISAS offers solutions to problems ranging from the digital exploration of traditional neuroanatomical atlases (like the Paxinos/Watson mouse brain atlas) to network analysis according to graph-theoretic measures and the direct incorporation of cell-type connectivity with computational simulation. Its broad feature set offers many solutions within a single common data neuroinformatics framework. *Golgi* may distinguish itself from neuroVIISAS by focusing narrowly on solving a single neuroinformatics problem very well: spatially exploring rat macroconnectivity. Constraining the use-case of our system allows us to constrain its User Interface and by proxy, the learning-curve that a user will face when using *Golgi*. This narrow approach is aligned with a trend in consumer-facing software products towards smaller, “one-solution” applications. This trend has two major causes. First, users seek out new applications to solve a narrow problem they face. As such, single-purpose apps tend to outcompete “all-in-one” apps because single-purpose apps better capture “first-to-mind” market positioning than do larger apps. Secondly, data aggregation, shared ontological frameworks, and common interchange formats like the NIFSTD allow single-focus neuroinformatics solutions to share data and resources while each maintaining a focused User Experience and minimized learning period.

The Mouse Connectome Project (MCP) produced by the Laboratory of Neural Imaging (Hintiryan et al., 2012) offers a user-friendly online web app for exploring macroconnectivity in the C57Bl/6J mouse brain. The approach taken by the MCP is unique to the approach taken by neuroVIISAS or BAMS: rather than aggregating and synthesizing connectivity data from the available corpus of literature (as did BAMS and neuroVIISAS), the MCP itself generated novel connectivity data using state-of-the-art double co-injection tract tracing techniques. The MCP makes this data available online in the form of both queryable tables of connectivity and an interactive display interface. This interface allows users to view coronal section slices of the actual microscopy data collected for each experiment corresponding to queried regions and connections of interest. Rather than display interactive coronal sections, *Golgi* distinguishes itself from the MCP by displaying the entire brain as an embryonic flatmap upon which several connections that may have spanned numerous serial sections can be contiguously displayed simultaneously. This approach allows for users to more intuitively grasp the spatial relationships between several connections at once than is afforded in a serial section viewer like the MCP.

The interactive neural map produced by Jianu et al. (2012) allows users to explore diffusion tensor imaging tractography data in two-dimensions via a web interface. Like the MCP, Jianu’s neural map displays connectivity data collected *de novo* explicitly for the neural map. Jainu’s map makes it easy for users to explore the relationships between white matter tracts (as observed in diffusion tensor imaging) and their relationship to one another in space. Yet unlike the MCP or neuroVIISAS, Jianu’s map is unable to provide the user with any information about macroconnections and the relationship between brain parts. While both Jainu’s neural map and *Golgi* take advantage of two-dimensional visualizations and brain flattening the two systems take a very different approach to the problem of flattening the brain. Jainu’s map simultaneously displays coronal, horizontal, and midsagital views in which all fiber tracts are simultaneously displayed across all three views. This means that, for example, two tracts viewed in a midsagital view, one more medial than the other, may appear to overlap across each other. This forces the user to reference the horizontal or coronal view to distinguish the relative mediolateral position of that tract. *Golgi* instead relies on the Swanson (2004) flatmap to show the brain as a single flattened surface. This decision lets us show the entire brain in a single view and (when combined with the neuroanatomical compass we provide) makes it easier for users to understand the spatial relationships between regions and their connections than would be accommodated in the three-view system used in Jainu’s neural map.

## Conclusion

We envision four threads of future technological growth for *Golgi*: data, display, detail, and development.

### Data

The data inside *Golgi* will be under pressures to grow in two directions: deep within a species and wide between species.

As more data within a species is collected experimentally or curated into knowledge management systems so too should *Golgi* include these new findings. To this end, establishing high-throughput pipelines for updating the data inside *Golgi* and designing API services between *Golgi* and other neuroinformatics tools will help ensure that *Golgi* expands its usefulness and is populated with contemporary data.

As a parallel line of development, incorporating different existing brain atlases within *Golgi* would increase the depth of available data for a given species. These atlases and their atlas-specific reports could exist independently from data registered to other atlases, or could be spatially co-registered for comparison across atlases for which strict topological relationships between terms and regions have been established (though this process comes with unique challenges of its own.)

As an orthogonal vector of growth, incorporating findings from other species into separate maps is rate-limited not only by its availability but by the existence of hierarchical, internally-consistent nomenclatures and corresponding reference atlases in which this data can be contextualized. To this extent, *Golgi’s* potential to represent data from other species depends on the neuroanatomy community’s progress in producing high-caliber, interoperable brain atlases and nomenclatures for more species. *Golgi’s* data architecture and front-end structure have been designed such that accommodating future brain atlases and atlases from different species can be done without drastically overhauling either the frontend or backend framework.

### Display

The way that connectomics data is displayed is constrained by the affordances allowed by our two-dimensional display devices. Decisions in *Golgi’s* construction—such as the choice of the Swanson (2004) embryonic flatmap as our atlas framework—were constrained by the fact that we predict our users will (overwhelmingly) use two-dimensional display devices and human-interface devices like a keyboard and mouse. Given these constraints, a two-dimensional map is well suited for a world of two-dimensional human-computer interaction.

As both human-computer interaction and display technologies evolve so too will our software. To this end, one major future developmental branch for *Golgi* will be to use new methods of displaying the nervous system. New visualization and interactions methods may compel us to—at minimum—reimagine how we display data on our existing brain atlases. At their most disruptive, new three-dimensional or four-dimensional (that is, those that incorporate how connectomic and proteomic data changes over the course of development, and during dynamic functioning) visualization techniques may require us to invent wholly new ways to visualize the nervous system.

### Detail

The state-of-the-art in connectomics is growing in precision and detail. *Golgi* currently renders assertions of connectivity at the macrolevel (between brain regions) due to the amount of macrolevel data available compared to mesolevel data (between cell populations). The connectomics community—in collaboration with neuroanatomists—must develop frameworks for distinguishing mesonodes (distinct cell populations) from one another before our existing macrolevel data can be interpreted in this new context (and new experiments can be performed de-novo at the mesolevel). As more connectivity information becomes available in mesolevel frameworks and mappable on mesolevel atlases, *Golgi* will need to provide affordances for displaying data at the mesoscopic level as well.

### Development

Beneath advancing the complexity of *Golgi’s* display interface, widening and deepening the level of connectomic data it contains, and increasing the scope of connectivity users can explore lies the quotidian albeit critical operational challenge of ensuring *Golgi’s* continued hosting, maintenance, and active development.

*Golgi’s* hosting on Amazon Web Services helps us balance the demands of keeping *Golgi* adequately available to interested users against the need for cost-efficiency. Amazon Web Services Elastic Cloud Compute service allows us to run *Golgi* cost-effectively when demand is low, and expand capacity accordingly only when demand is high. High server availability and routine machine image backups help us ensure that any unpredicted system failures can be quickly remediated with minimal system downtown and data loss.

*Golgi’s* data repository, as collected from BAMS, is currently being developed alongside a semi-automated daemon system that keeps *Golgi* up-to-date with BAMS without human intervention. This system, built on top the Kimono API prototype pipeline we initially built for populating *Golgi*, will periodically check for discrepancies between *Golgi’s* data and BAMS data and update *Golgi* to minimize these differences. This means that as BAMS grows and is populated with more and better connectional data, so too will *Golgi* grow along with it. This helps us focus available development labor toward more creative and productive ends in expanding *Golgi* than routine data aggregation.

Finally, we released *Golgi* under open-source public version control on GitHub. Now, any enthusiastic developer can learn more about neuroinformatics and freely contribute to the development of this tool for the betterment of the community and the state-of-the-art. This reflects not only our desire to spread *Golgi’s* adoption as far as we can, but also our belief that the best way to inure *Golgi* against deprecation and disinterest is to remove barriers to community involvement in *Golgi’s* development. This type of public stewardship of projects and ideas not only helps make *Golgi* better, it helps enfranchise talented, interested developers looking to join our fast-growing field.

## Author Contributions

Conceived and developed the project: RAB, LWS; Developed software: RAB; wrote the manuscript: RAB, LWS.

## Conflict of Interest Statement

The reviewers Marcus Kaiser and Sharon Crook declare that, despite being the Topic Editors of the Frontiers Research Topic this manuscript is part of, the review was handled objectively and no conflicts of interest exist. The authors declare that the research was conducted in the absence of any commercial or financial relationships that could be construed as a potential conflict of interest.
